# Supplementary material for: Are carbon emissions trading and green financial instruments synergistic? -Comprehensive quantitative research based on content analysis
Source: PLoS One. 2024 Mar 7;19(3):e0298601. doi: 10.1371/journal.pone.0298601 (PMC10919601; doi:10.1371/journal.pone.0298601)
Supplement: S1 Data — (ZIP) [file pone.0298601.s001.zip › Data/地级市Z_F工作报告.zip!/╕ú╜¿/╚2├≈╩╨/└┤╫╘╣1⁄2╓┌║┼í╛┬φ┐╦╩2╛▌═°í┐_2016.docx]

各位代表：

　　现在，我代表市人民政府向大会报告政府工作，请连同《三明市国民经济和社会发展第十三个五年规划纲要（草案）》一并审议，并请市政协各位委员和其他列席人员提出意见。

　　一、2015年和“十二五”时期工作回顾

　　过去一年，我市各级政府在省委、省政府和市委的正确领导下，深入贯彻习近平总书记系列重要讲话精神和对福建工作的重要指示，突出“念好发展经、画好山水画”工作主题，全力稳增长、调结构、促改革、惠民生，各项事业建设取得新成效。初步统计，全市生产总值1713.05亿元，增长8.5%；规模以上工业增加值866.5亿元，增长8.4%；地方一般公共预算收入93.68亿元，增长3%；固定资产投资1912.02亿元，增长19.3%；外贸出口117.7亿元，增长7.7%；实际利用外商直接投资1.56亿美元，增长11.4%；社会消费品零售总额444.47亿元，增长9.8%；城镇居民人均可支配收入27393元，增长8.7%；农村居民人均可支配收入12806元，增长9.8%；居民消费价格总水平上涨1.4%；节能减排年度任务可以完成。一些重要领域、重点项目实现突破：

　　——漳永高速、莆炎高速三明莘口至明溪城关段建成通车，莆炎高速永泰梧桐至尤溪中仙段、中心城市快速通道二期开工，南龙铁路加快建设，三明沙县机场完成竣工验收和行业验收。

　　——宁化月兔空调、台明离心球墨铸管、中科动力新能源汽车、永安锂电池石墨负极材料、通飞MS760飞机制造等重大项目落地或投产，三明高新技术产业开发区升格为国家级高新区，三明国家农业科技园区成功获批。

　　——我市荣获第四届全国文明城市，入闱全国小微企业创业创新基地城市示范，入选国家生态保护与建设示范区，列入国家中小城市综合改革试点。

　　——我市“医药、医保、医疗”联动改革做法写入中央“十三五”规划建议；国家公共文化服务体系示范区创建基本达标；成功承办首届全国青运会乒乓球赛事，三明籍运动员在举重世锦赛上获得3枚金牌，并打破世界记录。

　　一年来，主要工作和成效是：

　　（一）围绕稳定增长，深入贯彻“三比一看”。认真落实国家和省上稳增长政策，出台推动工业创新转型稳增长24条、做好地产品采购6条、优化金融服务7条等措施，助力企业加快发展。深入开展“敢担当、解难题、立新业”活动，50个市级重点难题已解决29个，1252个县级难题已解决1072个。启动“千名干部服务千家企业”活动，有效帮助首批230家企业解决资金、审批、销售、管理等问题。强化融资协调服务，设立20亿元产业发展基金、7.1亿元应急转贷资金，帮助155家企业协调解决信贷资金47.6亿元。制定实施进一步扩大有效投资的意见，175个重点增投项目完成投资379.8亿元、比年度计划增加76.3亿元，88个项目争取国家专项建设基金27.72亿元。新签约“三维”项目182个、总投资590.1亿元，与省属企业对接合作项目88个、总投资664.7亿元。

　　（二）围绕提质增效，加快产业转型升级。出台产业转型升级实施意见和实施《中国制造2025》行动方案，239个技改项目建成投产或部分投产，43个项目列入省级智能制造重点项目，沙县列入省级县（市、区）智能制造试点示范基地。传统产业加快改造提升，稀土、氟化工、生物医药及生物等新兴产业发展势头良好，高技术产业增加值增长19.3%。高优粮食、绿色林业、精致园艺、生态养殖、现代烟草等五大特色农业加快发展，新增设施农业5000亩，“中国稻种基地”获得国家和省上支持，市级以上农业龙头企业实现产值340亿元。现代服务业发展提速，50个重点项目建成或部分建成，文化产业主要指标增幅高于全省平均水平，游客接待量、旅游总收入分别增长13.8%和16.8%。“互联网+”新业态快速发展，5个县列入国家电子商务进农村综合示范县，全国首个县级下一代互联网产业试点基地落户沙县，全市电子商务交易额达到140亿元。启动“双创示范”工程，新建众创空间15家、孵化器4家。2人入选国家科技部第二批创新创业人才“推进计划”，2个团队、4人入选省第四批引进人才“百人计划”，新建6家院士专家工作站。

　　（三）围绕优化环境，统筹推进城乡建设。实施宜居环境建设项目673个，集中成片棚户区改造、市政设施提升、绿道网建设等工作取得新成效。抓好市区16个重中之重项目，城市绿道一二期全面建成，中心城区综合交通路网规划完成。生态新城加快建设，列入国家低碳城试点，湿地公园、三明北大附属实验学校、进口商品直销中心、平行进口汽车展销中心等项目落地。深入实施“大城关”战略，县城品位和特色进一步提升，20个重点小城镇改革发展取得新进展。加快美丽乡村建设，13个村列入国家旅游扶贫试点村，100个村列入省级乡村旅游特色村。积极创建国家级生态文明建设示范区，完成造林绿化35.1万亩、水土流失治理24.5万亩，明溪、建宁通过国家生态县技术评估，5个县入选中国百佳深呼吸小城。

　　（四）围绕激发活力，持续深化改革开放。开展“深化林改推进年”活动，新增新型林业经营组织673家，新发放林权抵押贷款16.5亿元，其中全国首创林权按揭贷款7.9亿元，我市被确定为全国集体林业综合改革试验示范区。推广沙县农村金融改革经验，设立112个村级融资担保基金，建成全省首个县级农村产权交易中心。稳步推进土地流转，新增土地流转面积1万亩。出台扶持企业利用资本市场加快发展的办法，科飞新材、文鑫莲业、奥翔塑胶等3家企业在“新三板”上市，永安林业、青山纸业等上市企业再融资取得突破，市城投公司10亿元中期票据获批发行。厦门银行、光大银行三明分行开业。实施“三证合一、一照一码”登记制度，新增各类市场主体2.55万户。积极承接福建自贸区“外溢”效应，“陆地港”保税仓库建成运营。区域协作不断加强，成功举办第十一届林博会，明港澳台侨合作进一步深化。

　　（五）围绕和谐稳定，大力发展社会事业。民生支出比上年增长22.3%，22项为民办实事项目全面完成。全市新增城镇就业2.83万人，农村劳动力转移就业4.54万人，城镇登记失业率2.36%。城乡居民养老保险基础养老金、城镇企业退休人员养老待遇、城乡低保和农村“五保”补助标准进一步提高，机关事业单位养老保险制度改革有序推进。保障性安居工程新开工5478套，基本建成9180套，超额完成年度任务。扶贫开发加快推进，“348”精准扶贫工作机制在全省推广，完成造福工程搬迁和危房改造3.18万人，解决饮水不安全人口6.87万人，全年减少贫困人口2.5万人。全市乡镇（街道）公办幼儿园实现全覆盖，全面完成义务教育发展基本均衡县创建任务。在全省率先推进现代医院管理制度、医养结合试点、医保政策城乡一体化，群众看病负担进一步减轻，医院收入结构更加优化。计生服务水平稳步提高，在省内率先建立全市计生社会诚信体系。万寿岩国家考古遗址公园获得中央专项资金补助，文化“六联六创”工作机制在全省推广。三明市第六届运动会顺利举办。创新社会综合治理，强化信访工作，抓好安全生产，有效保障食品药品安全，社会保持安定稳定。村（居）委会换届工作顺利完成。老龄、工会、青少年、妇女儿童、残疾人工作扎实开展，司法行政、统计、科普、民族宗教、档案、地方志、水文、气象、防震减灾、库区移民工作取得新进展。双拥共建和人民防空、民兵预备役、国防后备力量建设得到加强。

　　（六）围绕“三严三实”，强化法治政府建设。深入开展“三严三实”专题教育，严格落实中央八项规定精神，政府工作作风持续改进。坚持依法行政，自觉接受人大、政协监督，人大代表建议和政协提案办理质量进一步提高，政府与法院、检察院、工会互动联系机制不断完善。深化简政放权，全面完成市、县政府机构改革，编制并公布行政审批项目清单、公共服务项目清单、权力清单、责任清单。推行投资项目全程代办服务，建成三明市网上办事大厅，中介服务中心建设进一步规范。加强政府绩效管理，加大效能督查力度，深化政务公开，强化行政监察和审计监督，勤政廉政建设取得新成效。

　　各位代表，2015年工作任务的完成，为“十二五”规划画上了圆满句号。经过五年的艰苦努力，我市经济社会发展跃上新台阶。这五年，是三明经济实力显著增强的五年，是城乡面貌发生重大变化的五年，是对外影响大幅提升的五年，也是人民生活明显改善的五年。在经济实力上，全市生产总值年均增长11.1%，人均生产总值突破1万美元大关；一般公共预算总收入超过130亿元，累计完成全社会固定资产投资是“十一五”时期的2.6倍；冶金及压延、装备制造、林产加工、矿产加工、纺织、生物医药及生物等6条特色产业链产值突破2000亿元，现代农业产值突破1000亿元，三次产业比例从17.3∶49.2∶33.5调整为14.7∶51.1∶34.2。在城乡面貌上，全市常住人口城镇化率从51.1%提高到56.1%；市区建成区面积从27.85平方公里拓展到37.45平方公里；我市入闱全国生态城市竞争力十强，获得省级生态市称号；动车从无到有，新增永宁、建泰、漳永、莆炎等4条高速公路，提前实现县县通高速，集高速公路、快速铁路、机场于一体的现代化交通体系基本形成。在对外影响上，一批国家级试点、试验区落户我市，林改、医改、农村金融改革等品牌在全国全省打响，文明城市、综治“长安杯”、未成年人思想道德建设工作先进城市等国家级荣誉成功获得，世界客属第25届恳亲大会和第七届海峡两岸高峰论坛成功举办。在人民生活上，城乡居民人均可支配收入年均分别增长11.1%、13%；五年城镇新增就业13.97万人、农村劳动力转移就业24.56万人，累计新建保障性安居工程4.4万套、发放租赁补贴4084户，建立城乡低保、农村“五保”供养标准自然增长机制；全面实现“双高普九”，累计发放各类助学资金4.3亿元，受益学生105.38万人次；五年卫生基础设施投入10.13亿元，人均基本公共卫生服务经费从15元增加到40元；新建市区“六馆一场一宫”，县、乡、村文化设施基本达标，实现全市行政村农民体育健身工程全覆盖。

　　各位代表，“十二五”发展成就来之不易，这是在省委、省政府和市委正确领导下，在历届政府打下的良好基础上，经过全市上下奋力拼搏取得的。在此，我代表市人民政府，向全市人民，向人大代表、政协委员、各民主党派、工商联、各人民团体、无党派人士、离退休老同志和社会各界人士，向中央和省属驻明单位、驻明部队、武警官兵、政法干警，向关心支持三明发展的港澳台同胞、海外侨胞、国际友人，致以衷心的感谢和崇高的敬意！

　　在看到成绩的同时，我们也清醒地认识到存在的困难和问题，主要是：经济下行压力依然较大，一些经济指标增速放缓，部分产业和企业比较困难，财政增收压力较大，金融风险隐患增多；产业转型升级任务重，传统产业占比较大，新兴产业规模偏小，自主创新能力亟需加强；城市规划、建设、管理有待提升，中心城市辐射带动力不强，城镇集聚人口能力不足；社会保障水平较低，教育、文化、卫生等公共服务水平与群众期望还有差距，扶贫开发任务比较艰巨；节能减排压力较大，公共安全还面临一些突出问题；一些干部担当意识、服务意识不强，不作为、慢作为、乱作为的问题不同程度存在。对此，我们要高度重视，采取有力措施加以解决。

　　二、“十三五”时期主要目标和任务

　　“十三五”时期，是全面建成小康社会的决胜阶段。综观“十三五”时期国内外发展大势，三明既面临难得的发展机遇，又面临严峻的挑战，但总体上机遇大于挑战。我们要准确把握发展机遇内涵的深刻变化，抢抓机遇、乘势而上、真抓实干，不断开创经济社会发展新局面。

　　根据市委八届十一次全会通过的“十三五”规划《建议》，市政府组织编制“十三五”规划《纲要》（草案），明确我市“十三五”发展的指导思想是：高举中国特色社会主义伟大旗帜，全面贯彻党的十八大和十八届三中、四中、五中全会精神，以马克思列宁主义、毛泽东思想、邓小平理论、“三个代表”重要思想、科学发展观为指导，深入贯彻落实习近平总书记系列重要讲话精神和对福建工作的重要指示，坚持“四个全面”战略布局，坚持发展是第一要务，着力创新发展、协调发展、绿色发展、开放发展、共享发展。按照省委省政府决策部署，牢牢把握中央支持海峡西岸经济区建设和福建加快发展的重大机遇，突出“念好发展经、画好山水画”工作主题，以保持经济稳定较快增长为目标，以转型升级为主线，以提高发展质量和效益为中心，加快形成引领经济发展新常态的体制机制和发展方式，全面推进经济建设、政治建设、文化建设、社会建设、生态文明建设和党的建设，推动经济社会发展再上一个新台阶，努力为建设机制活、产业优、百姓富、生态美的新福建作出新贡献。主要目标是：综合实力持续提升，地区生产总值年均增长9%左右，提前实现地区生产总值和城乡居民人均收入“两个翻番”，到2020年人均生产总值达到10万元，城乡居民人均可支配收入分别突破4万元和2万元，一般公共预算总收入达到180亿元；城乡区域更加协调，新型城镇化质量明显改善，户籍人口城镇化率达到50%左右，常住人口城镇化率达到64%左右；改革开放全面推进，重点领域和关键环节改革取得突破，对内对外开放的广度深度不断拓展；创新创业活力增强，自主创新能力进一步增强，高新技术产业增加值占GDP的比重达12%；生态优势持续凸显，生态环境质量继续保持全省领先，森林覆盖率保持在73.87%以上；人民生活更加幸福，社会保障体系进一步完善，基本公共服务进一步均等化，到2018年现行国定扶贫标准对象全部脱贫，到2020年省定扶贫标准对象全部脱贫，5个省级扶贫开发工作重点县全部脱帽。主要任务是：一是打造产业转型升级版；二是培育经济发展新动力；三是推进体制机制改革创新；四是建设国家扶贫改革试验区；五是推动城乡区域协调发展；六是完善现代基础设施体系；七是创建生态文明先行示范区；八是深化全方位开放合作；九是构建国家公共文化服务体系示范区；十是强化民生保障和社会福祉；十一是加强法治三明建设。

　　今后五年，市政府将团结带领全市人民，聚焦全面建成小康社会，牢固树立和贯彻落实五大发展理念，围绕“十三五”目标要求和工作任务，聚精会神搞建设、一心一意谋发展，高质量、高水平落实“十三五”规划，为今后更长时期发展打下坚实基础、提供有力支撑，进入更有质量、更加协调、更可持续的新的发展阶段。

　　三、2016年政府主要工作安排

　　今年是“十三五”开局之年。我们要按照省委、省政府和市委的部署，积极适应新常态，坚持稳中求进总基调，着力加强供给侧结构性改革，突出做好稳增长、调结构、强动力、惠民生、防风险，努力保持经济平稳较快发展，确保“十三五”发展开好局起好步。全市经济社会发展主要预期目标是：地区生产总值增长9%左右；地方一般公共预算收入增长5%；固定资产投资增长20%；外贸出口增长3%，实际利用外商直接投资增长6%；城镇登记失业率控制在3.8%以内；社会消费品零售总额增长12%，居民消费价格总水平涨幅控制在3%左右；城镇居民人均可支配收入增长9.5%，农村居民人均可支配收入增长10%；完成单位生产总值能耗降低和主要污染物减排年度任务。围绕上述目标，着重抓好以下七个方面工作：

　　（一）着力推进转型升级，提高有效供给能力

　　做强先进制造业。注重从供给侧发力，对接省上制造业升级行动，落实扶持工业发展政策措施，支持企业开拓市场，有效降低实体经济企业成本，提高企业市场竞争力。推动冶金及压延、汽车及机械装备、林产加工、纺织等传统产业转型升级，鼓励龙头企业增资扩股、兼并重组，促进产业集聚延伸、提质增效，力争传统产业实现产值1700亿元以上。实施新一轮技术改造，抓好300项以上重点技改项目，力争建成投产或部分投产200项以上。推进“两化”深度融合，实施“数控一代”“智能一代”工程，完成“机器换工”100台（套）以上，培育市级智能制造示范企业30家以上。发展壮大新材料、新能源、节能环保、生物医药及生物等新兴产业，加快推进三农氟化工二期、稀土分离厂、锂离子正极材料二期、永安石墨（烯）材料、新能源汽车等项目，力争战略性新兴产业实现产值600亿元以上。加大矿产资源整合力度，引导矿产开发“三集中、三延伸”，推动资源优势转化为产业优势。

　　提升现代服务业。大力发展生产性服务业，促进现代物流、服务外包、金融服务、融资租赁、信息技术、研发设计等服务业与制造业有机融合。深入实施“互联网+”行动计划，加快电子商务发展，抓好电商产业园建设，支持20家年销售额超亿元的电商企业发展，培育一批覆盖全产业链的行业垂直电商平台和第三方电商，拓展农村电子商务。扶持文化产业园区建设和龙头企业发展，做大文化创意、工艺美术等产业。发展壮大旅游业，支持泰宁创建国家全域化旅游示范区，加快开发三明郊野国家地质公园，大力发展特色民宿等乡村旅游，成立三明品牌景区营销中心，推进旅游产品向观光、休闲、度假、安养并重转变。繁荣商贸市场，加快万达广场等商业综合体建设，实施一批重点专业市场项目，培育壮大消费新业态。积极发展健康、健身、家政等服务业，大力发展居家养老、社区养老、机构养老等多样化养老服务。加大房地产去库存力度，支持首套房、首改房刚需购房，鼓励商业地产转型，发展住房租赁市场，促进房地产业健康发展。

　　强化转型要素支撑。发挥产业发展基金引导作用，推动各类创新要素向企业集聚、为企业服务。加快国家级高新区和省级重点园区建设，鼓励开展“二次招商”。推进小微企业创业创新基地城市示范建设，新增众创空间和孵化器15家以上，新增小微企业4000家以上。深化与清华、北大等高校产学研合作，建好中机院海西分院等国字号研究机构，实施70个以上重大科技项目，新增省级工程技术研究中心、重点实验室、技术公共服务平台10家以上，新建院士专家工作站5家以上。深入实施质量强市战略，支持企业创建品牌和制修订行业标准。加强知识产权保护和运用。创新人才工作机制，落实“海纳百川”高端人才聚集计划，大力培育本土人才，吸引更多优秀人才和创新团队在我市创业发展。

　　（二）着力强化项目建设，积极扩大有效投资

　　加强基础设施建设。交通方面，加快南龙铁路和三明南站、永安南站站房及周边配套设施建设，全线开工兴泉铁路、浦梅铁路建宁至冠豸山段，实施鹰厦线台江至荆西段改线工程；加快厦沙高速三明段和莆炎高速永泰梧桐至尤溪中仙段建设，力争开工莆炎高速尤溪中仙至三元莘口、明溪至建宁段，完成机场等4个互通及连接线工程，续建和新开工国省干线180公里。能源方面，加快建设海西天然气管网三明段一期、泰宁池潭电厂扩建等项目，开工建设大唐国际沙县热电联产、宁化风电等项目，深化三明核电、抽水蓄能电站等项目前期工作；建成三明安居、沙县镇头两座220千伏变电站，推进农村电网改造升级工程。城市公用设施方面，实施新一批市政提升“五千工程”，加快充电桩（站）建设，推进市区南部新城地下综合管廊建设，完成列东大桥改造工程，市区第二供水工程建成投用。水利方面，加快尤溪兴头、沙县双溪、永安溪源、明溪黄沙坑等中型水库及烟基水源工程建设，推进闽江上游重点流域防洪工程，抓好万里安全生态水系建设。通信方面，推进“数字三明·宽带工程”和无线城市建设。

　　加快实施重点项目。着力抓好464个列入省行动计划重大项目，完成投资460亿元以上，新开工20个以上，建成或部分建成70个以上。实施470个市级以上重点项目，完成投资580亿元以上，确保每个县（市、区）新开工、建成或部分建成项目各10个以上。持续深化与中机院、中节能、中纺院等央企合作，跟进落实已签约民企、外企和省属企业项目，保持“月月有对接、月月有落地”。抓好国家专项建设基金项目争取和建设，继续实施一批增投项目和经营性项目，充分发挥国有资本投资引导作用。

　　完善项目推进机制。深化项目前期工作，加强项目策划储备和动态管理，实现项目持续滚动发展。健全重点项目一月一协调、一季一督查和市领导挂包、部门联动机制，及时解决存在困难和问题。积极示范推广PPP模式，完善政银企对接机制，全年新增贷款120亿元以上。推进和谐征迁，有效落实用地、用林等要素保障。优化审批服务，推行网上审批、并联审批和项目全程代办，促进项目早落地、早开工。

　　（三）着力抓好现代农业，加快精准扶贫开发

　　大力发展特色农业。严格保护耕地，加快粮食产能区和高产示范片建设，落实和完善农业补贴政策，确保粮食播种面积稳定在320万亩、总产稳定在110万吨以上。强化“一区三园”建设，加大用电等政策扶持，培育和引进一批农业龙头企业。大力发展设施农业、生态农业、智慧农业，持续做强五大特色优势产业和20条特色农业产业链，积极发展林下经济。推行“互联网+农产品营销”“定制农业”等新型组织方式，扩大农产品销售。加强农业“五新”推广，加快“中国稻种基地”建设，提高农业机械化水平。抓好“三品一标”认证，严格农产品质量安全监管，加强重大动植物疫病防控。

　　创新农业经营方式。培育壮大新型农业经营主体，加大对农民合作社、家庭农场、专业大户、龙头企业的扶持力度，新增市级以上示范农民合作社、示范家庭农场各50家，培育一批新型职业农民。全面推进农村土地承包经营权确权登记颁证工作。创新土地流转和规模经营方式，加快县级土地流转服务中心建设，推广土地股份合作、土地托管、土地信托流转等经营模式。积极发展农业经营性服务组织，完善农业社会化服务。

　　深入推进脱贫攻坚。加快三明国家扶贫改革试验区建设，突出5个省级扶贫开发重点县，统筹用好老区苏区政策和扶贫开发专项资金，全面深化“348”精准扶贫工作机制，推进精准扶贫“九个一批”，实施精准扶贫“五项工程”“三大行动”，落实精准扶贫“新八种模式”，实现年度脱贫2万人。实施造福工程搬迁1.5万人以上，建设集中安置区20个以上。抓好农村饮水安全巩固提升工程。加快少数民族乡村发展。做好库区移民安置和后期扶持。

　　（四）着力优化城乡建设，打造更加美好家园

　　做大做强城镇。创新规划理念，优化专项规划和控制性详规，加强城市设计，推进“多规合一”，提高城市规划水平。加快中心城市建设，抓好生态新城国家低碳城试点工作，抓紧实施中心城市快速通道二期、205国道市区过境段、306省道改线工程，建设梅列陈大至沙县富口、小蕉道路改线工程、万达至新行政服务中心等8条道路，推动市区与沙县、永安同城化取得新突破。加快市区两级园区融合发展，抓好梅列工业新城、吉口新兴产业园、荆西智能机械装备产业园、黄砂新材料循环经济产业园等重点园区建设，推进洋溪、贵溪洋、徐碧、台江、富兴堡等重点板块开发，继续实施沙溪十里闽学文化长廊、绿道三期等重点项目，提升市区经济实力和城市品位。坚持以城促产、强产兴城，突出“一县一园区”，做大“一县一业”，壮大县域特色经济。推动永安、大田分别建设国家级和省级新型城镇化试点，深化尤溪洋中镇省级“小城市”培育试点，抓好省市级重点乡镇改革发展。加快户籍制度改革，全面实施居住证制度，引导人口优先向城市和建制镇转移。

　　建设宜居环境。继续实施宜居环境建设行动计划，延伸拓展“点线面”攻坚，提升城镇“三边三节点”整治水平。创新村庄建设与治理模式，深入开展农村生活污水垃圾治理行动，整治村庄130个以上，创建省级美丽乡村示范村10个以上。加大历史文化名镇名村、历史建筑、传统村落保护力度。完善城市路网结构和优化公交线路，新建公共停车泊位1500个以上，有效缓解出行难、停车难等问题。强化城市精细化管理，推进综合执法，推广“街长制”“微城管”，继续深化“两违”综合治理。加强社区建设，推进老旧社区改造，规范提升物业管理水平。

　　加强生态建设。坚持绿色低碳循环发展，深化南方林区生态文明建设综合配套改革，抓好国家级生态文明建设示范区创建工作，力争3个县通过国家生态县技术评估，建成国家森林城市。科学划定生态保护红线，实施山水林田湖生态保护和修复工程，完成造林绿化和森林经营236.9万亩，综合治理水土流失24.5万亩。深化工业污染治理，完善园区污水处理设施，实施50项重点节能和循环经济项目，推进一批重点脱硫、脱硝设施升级改造工程。深化流域水环境综合整治，贯彻省河道保护管理条例，全面落实“河长制”，强化畜禽养殖面源污染治理，加强饮用水源地保护。开展燃煤锅炉、扬尘粉尘、机动车尾气等专项整治，加大黄标车淘汰力度。严格落实环保监管“一岗双责”，严查环境违法行为，实行生态环境损害赔偿。

　　（五）着力深化改革开放，增强发展动力活力

　　突破重点领域改革。深化集体林权制度改革，发展新型林业经营组织，拓宽林业融资渠道，设立三明林权交易中心，力争新型林业经营组织覆盖面达50%以上、新增林权抵押贷款15亿元以上。深化医药卫生体制改革，坚持“三医”联动，探索医养结合发展新路子，全面实行医用耗材（检验试剂）联合限价采购，理顺医疗服务价格，落实分级诊疗和双向转诊制度，争创国家中医药发展综合改革试验区。深化沙县农村金融改革，推广村级融资担保基金等做法，发展普惠制金融。继续实施“引银入明”工程，新引进2家以上区外银行。推动企业上市，支持企业在“新三板”、海峡股权交易中心挂牌交易，鼓励企业发行短期融资券、中期票据、项目收益债券，做好城投、国投、交建等公司发债工作，鼓励发展创业投资、风险投资。加强金融风险防范和化解，规范民间融资行为，依法处置信用违约，严守金融风险底线。深化工商登记制度改革，扩大“三证合一、一照一码”改革成果。抓好财政体制改革，整合不同部门管理的同类资金，稳步推进营改增扩围工作，加强地方政府性债务管理，推进政府购买服务工作。完成公务用车制度改革。

　　加大对外开放力度。主动对接“一带一路”战略，推动钢材、水泥、纺织、汽车及机械装备等优势产能“走出去”。积极复制推广自贸区试验创新成果，加强与沿海口岸通关协作，抓好平行进口汽车展销中心等项目建设，完善“陆地港”口岸和保税仓储功能，推进机场空港规划建设。加快培育一批出口基地和龙头企业，促进生产型企业出口稳定增长。用好林博会、台湾农民创业园、朱子文化园、宁化石壁海峡两岸交流基地等平台，深化明台经贸、文化、科技等领域交流合作。加强区域协作，密切与港澳地区、国外友城的沟通联系，做好与海外华侨华人及其社团的联络工作。

　　（六）着力保障改善民生，共享改革发展成果

　　提高社会保障水平。完善创业就业政策，统筹做好大中专毕业生、就业困难人员、农村转移劳动力、退役军人等就业工作，新增城镇就业2.2万人以上、农村劳动力转移就业2.6万人以上。扩大全民参保登记试点范围，全面推进机关事业单位养老保险制度改革，完善城乡居民基本养老保险制度，提高城乡低保、特困人员供养标准，全面实施临时救助制度。加大棚户区、危旧房改造力度，新开工保障性安居工程4800套，基本建成3500套。加强农村留守儿童、空巢老人、妇女、残疾人关爱服务，做好关心下一代工作，重视发展社会福利、红十字会、慈善、老龄事业。办好省市为民办实事项目。

　　加快社会事业发展。继续实施第二期学前教育三年行动计划，新建和改扩建一批公办幼儿园，支持普惠性民办幼儿园发展。启动“教育强县”创建工作，继续实施农村义务教育薄弱学校改造、城区中小学扩容工程等项目，促进普通高中优质特色发展。支持三明学院向应用型院校转变，争创国家、省示范性应用型本科高校。推动三明职业技术学院、福建水利电力职业技术学院等中高职院校创建省示范性现代职业院校，开展现代学徒制教育试点，加快培养一批中高级产业技术人才和紧缺急需人才，建立区域性职教联盟，支持校企共建实训基地，建成市职教园区。推进继续教育、特殊教育、民族教育和老年教育加快发展。深化“六联六创”服务方式，确保国家公共文化服务体系示范区创建成功。加强文化遗产保护利用，加快万寿岩国家考古遗址公园等重点项目建设。推进三歌文化传媒等院团整合改革。鼓励文化精品创作，繁荣发展社会科学、文学艺术事业。完善基层公共卫生服务体系，加强儿科、产科等专科建设，提升疾病防控能力。加强科普工作，提高全民科学素质。优化计划生育管理和服务，全面实施两孩政策，促进人口均衡发展。深入开展全民健身运动，积极发展老年体育，培养竞技体育后备人才，建成市少体校新校区。推进企业入驻“正统网”，开展第三次全国农业普查。做好档案和第二轮修志工作。

　　创新社会综合治理。完善群众性精神文明创建活动长效机制，巩固提升全国文明城市和未成年人思想道德建设工作先进城市创建成果。深化“平安三明”建设，推进城乡社区服务管理网格化，完善立体化治安防控体系，扎实做好反恐维稳工作。健全社会稳定风险评估机制，加强矛盾纠纷排查化解，依法处理信访事项。抓好重点领域安全生产专项治理，推进安全生产标准化建设。强化食品药品安全监管，完善从田头到餐桌全程管控体系。加强应急处置能力建设，提高防灾减灾水平。密切与各民主党派、工商联、无党派代表人士的联系与协商，支持工会、共青团、妇联等群团组织发挥更大作用，做好外事侨务、民族宗教工作。深化双拥共建，加强国防动员、国防后备力量和人民防空建设，推进军民深度融合发展。

　　（七）着力加强自身建设，提升政府服务效能

　　坚持依法行政。落实法治政府建设实施纲要，把政府工作全面纳入法治轨道。依法履行立法职权，加强立法能力建设。全面推进政务公开，完善政府信息公开制度。自觉接受人大及其常委会的法律监督、工作监督和政协的民主监督，认真办理人大代表建议和政协提案。推进依法决策，健全重大决策合法性审查机制，加强行政复议、行政应诉。严格公正文明执法，规范行政执法行为。进一步转变政府职能，持续深化简政放权和行政服务标准化建设，认真执行“四张清单”制度，规范提升中介服务水平，整合构建统一开放的公共资源交易平台。深入实施“七五”普法，落实国家工作人员学法用法制度，推进公共法律服务体系建设，引导全社会尊法学法守法用法。

　　深化作风建设。持续反对“四风”，自觉践行“三严三实”要求。坚持“四下基层”，继续开展“千名干部服务千家企业”活动，切实帮助基层、企业和群众解决实际问题。强化责任担当，勇于攻坚克难，深入开展“敢担当、解难题、立新业”活动，突破难点、消除痛点、打通堵点。落实“马上就办”，事情定了就办、办就办好，绝不允许拖拖拉拉、半途而废。加强效能建设，加大问责力度，坚决纠正不作为、乱作为，坚决整治“庸懒散拖”和为官不为等问题。

　　保持清正廉洁。严守党的政治纪律和政治规矩，严格执行《中国共产党廉洁自律准则》、《中国共产党纪律处分条例》和中央八项规定精神，始终把纪律和规矩挺在前面。严格落实党风廉政建设责任制，强化对重点领域、关键环节权力运行制约监督，用制度管权、管钱、管事。坚持厉行勤俭节约，反对铺张浪费，严控“三公”经费支出，实行预算公开透明。加大行政监察和审计监督力度，从严查处违法违纪案件，用严的制度和铁的纪律打造过硬干部队伍。

　　各位代表，蓝图催人奋进，圆梦需要实干。让我们更加紧密地团结在以习近平同志为总书记的党中央周围，在市委的坚强领导下，紧紧依靠全市人民，解放思想、脚踏实地、开拓进取，为实现全面建成小康社会目标而努力奋斗!
